# Molecular investigation of tick-borne pathogens in ticks removed from tick-bitten humans in the southwestern region of the Republic of Korea

**DOI:** 10.1371/journal.pone.0252992

**Published:** 2021-06-15

**Authors:** Mi Seon Bang, Choon-Mee Kim, Sang-Hyun Pyun, Dong-Min Kim, Na Ra Yun

**Affiliations:** 1 Department of Medical Science, College of Medicine, Chosun University, Gwangju, Republic of Korea; 2 Premedical Science, College of Medicine, Chosun University, Gwangju, Republic of Korea; 3 Graduate School of Chosun University, Gwangju, Republic of Korea; 4 Department of Internal Medicine, College of Medicine, Chosun University, Gwangju, Republic of Korea; University of Zambia, ZAMBIA

## Abstract

In this study, we investigated the presence of tick-borne pathogens in ticks removed from tick-bitten humans in the southwestern provinces of the Republic of Korea (ROK). We identified 33 ticks from three tick species, namely *Amblyomma testudinarium* (60.6%), *Haemaphysalis longicornis* (27.3%), and *Ixodes nipponensis* (12.1%) in order of occurrence via morphology and 16S rDNA-targeting polymerase chain reaction (PCR). Tick-borne pathogens were detected in 16 ticks using pathogen-specific PCR. From the results, 12 ticks (36.4%) tested positive for spotted fever group (SFG) *Rickettsia*: *Rickettsia monacensis* (1/12), *R*. *tamurae* (8/12), and *Candidatus* Rickettsia jingxinensis (3/12). Three ticks (9.1%) were positive for *Anaplasma phagocytophilum*. In addition, three ticks (9.1%) tested positive for *Babesia gibsoni* (1/3) and *B*. *microti* (2/3). In conclusion, we identified three tick species; the most common species was *A*. *testudinarium*, followed by *H*. *longicornis* and *I*. *nipponensis*. SFG *Rickettsia*, *A*. *phagocytophilum*, and *Babesia* spp. were the most frequently detected pathogens in ticks removed from tick-bitten humans. To our knowledge, this is the first report of *R*. *tamurae* and *Ca*. R. jingxinensis detection in Korea. The present results will contribute to the understanding of tick-borne infections in animals and humans in the ROK.

## Introduction

Ticks are major vectors of pathogens, such as bacteria, viruses, and protozoans. These arthropods and protozoans can transmit a variety of diseases in humans and animals [1]. Tick-borne diseases are caused by viral or bacterial pathogens transmitted through tick bites. Several tick-borne diseases, such as Lyme disease (caused by *Borrelia* spp.), spotted fever group rickettsioses (caused by *Rickettsia* spp.), anaplasmosis (caused by *Anaplasma phagocytophilum*), bartonellosis (caused by *Bartonella* spp.), Q fever (caused by *Coxiella burnetii*), and babesiosis (caused by *Babesia* spp.) have been reported in the Republic of Korea (ROK) [2].

The incidence of tick-borne diseases in the ROK is increasing due to global warming, increased conduct of outdoor activities, and increased international travel. The growing number of tick bites each year poses an escalating risk of tick-borne diseases [2]. A tick survey conducted by the Korea Centers for Disease Control and Prevention (KCDC) from 2013 to 2015 reported that, of the 29,992 ticks collected from 29 sites, *Haemaphysalis longicornis* and *H*. *flava* were distributed nationwide, while *Amblyomma testudinarium* was predominantly located in the southwestern area of the ROK [2]. The geographical distribution of chigger mites is mainly distributed in the area where scrub typhus is prevalent [3]. However, few studies have investigated the prevalence of tick-borne pathogens in ticks removed from tick-bitten humans in the ROK. Therefore, it is necessary to determine the extent of tick-borne pathogens in the ROK and to characterize them.

The present study aimed to investigate the presence of tick-borne pathogens in ticks removed from humans in the southwest provinces of the ROK. Our study detected the DNA of tick-borne pathogens in ticks using pathogen-specific nested PCR. The results of this study will contribute to the understanding of the interactions between ticks and pathogens that cause diseases in humans. Additionally, this study could assist in describing the potential for human tick attachment to the public health practitioners of ROK.

## Materials and methods

### Ethics statement

This study was approved by the Ethics in Human Research Committee of Chosun University Hospital under an institutional review board (IRB), which approved all the experiments that used ticks removed from tick-bitten humans (approval no. CHOSUN NON2019-001). The IRB approved the protocol with verbal consent for the use of ticks and not human subjects. We have read and explained the verbal version of the consent form to use samples for research purposes and obtained individual consent.

### Tick samples

Ticks were removed by individuals or by the physicians in Gwangju Metropolitan City and the province of Jeollanam, which are in the southwest provinces of the ROK, between March 2014 and September 2017 (S1 Fig). Ticks were collected from patients visiting Chosun University Hospital located in this area. All ticks were morphologically identified according to species and life stage using a microscope and standard taxonomic keys [4]. The ticks were washed in 70% ethanol, rinsed twice with sterile phosphate-buffered saline (PBS), added to a hard tissue grinding MK28 tube (Bertin Technology, Rockville, MD, USA) containing 800 μL of PBS with 1× PC/SM (penicillin and streptomycin), ground using a FastPrep^®^-24 Classic instrument (MP Biomedicals, Solon, OH, USA), and stored at -80 °C until used for DNA extraction.

### DNA extraction

We mixed 150 μL of the ground tick with 150 μL ATL (animal tissue lysis solution) buffer and 20 μL proteinase K, incubated at 56 °C overnight for lysis, and genomic DNA was extracted using a QIAamp Tissue & Blood Mini Kit (Qiagen, Hilden, Germany) according to the manufacturer’s instructions. DNA was eluted into 50 μL TE buffer and stored at -20 °C until PCR amplification.

### Polymerase Chain Reaction (PCR)

To detect the presence of *Rickettsia* DNA, the outer membrane protein A gene (*ompA*), citrate synthase gene (*gltA*), and the 17 kDa protein gene (*17 kDa*) of the spotted fever group *Rickettsia* species were targeted. The heat shock protein gene (*groEL*) and ankyrin-related protein gene (*ankA*) were used to detect *A*. *phagocytophilum*. To detect the presence of *Borrelia* DNA, the CTP synthase gene (*pyrG*) was targeted. The 16S-23S internal transcribed spacer region (*ITS*) was used to detect *Bartonella* species. The *htpAB*-associated repetitive element *IS1111* was used to detect *Coxiella* species. To detect the presence of *Babesia* species, the sequence of its 18S rDNA was targeted. To identify tick species, conventional PCR targeting of the mitochondrial 16S rRNA gene (16S rDNA) was performed. All PCR primers used for detecting tick-borne pathogens, PCR conditions, and the corresponding product sizes, are listed in Table 1. Conventional PCR (C-PCR) was performed in 20 μL reaction volumes using AccuPowerR PCR PreMix (Bioneer Corp., Korea). Each PCR mixture consisted of 16 μL of distilled water, 1 μL of each primer (10 pmol/μL), and 2 μL of genomic DNA as the template. For 16S rDNA C-PCR and 18S rDNA nested PCR (N-PCR), we performed PCR using AmpliTaq Gold 360 Master Mix (Applied Biosystems, CA, USA) instead of AccuPowerR PCR PreMix.

**Table 1:** Oligonucleotide primers and PCR conditions used for the detection of tick-borne pathogens in ticks removed from tick-bitten humans.

Identification	Target gene^a^	Primer name	Nucleotide sequence (5’-3’)	Product size (bp)	PCR conditions (°C/sec)				Reference
					Denaturation	Annealing	Extension	Cycles	
*Rickettsia* species	*ompA*	RR190.70F	ATGGCGAATATTTCTCCAAAAA	634	94/30	50/30	72/60	40	[5, 6]
		RR190.701R	GTTCCGTTAATGGCAGCATCT						
		RR190.70F	ATGGCGAATATTTCTCCAAAAA	535	94/30	50/30	72/30	5	[5]
		RR190.602R	AGTGCAGCATTCGCTCCCCCT		94/30	54/30	72/30	30	
	*gltA*	GLTA1F	GACGGTGATAAAGGAATCTTG	1022	95/20	47/30	72/60	40	[7]
		GLTA1R	CATTTCTTTCCATTGTGCCATC						
		GLTA2F	CTACGAACTTACCGCTATTAG	446	95/20	43/30	72/30	5	
		GLTA2R	GACCAAAACCCATTAACCTAAAC		95/20	48/30	72/30	30	
	*17 kDa*	Rr17k.1p	TTTACAAAATTCTAAAAACCAT	539	95/30	57/60	72/120	35	[8]
		Rr17k.539n	TCAATTCACAACTTGCCATT						
		Rr17k.90p	GCTCTTGCAACTTCTATGTT	450	95/30	57/60	72/120	35	
		Rr17k.539n	TCAATTCACAACTTGCCATT						
*Anaplasma phagocytophilum*	*groEL*	GRO607F	GAAGATGCWGTWGGWTGTACKGC	688	95/30	54/30	72/60	30	[9]
		GRO1294R	AGMGCTTCWCCTTCWACRTCYTC						
		GRO677F	ATTACTCAGAGTGCTTCTCARTG	445	95/30	57/30	72/60	30	
		GRO1121R	TGCATACCRTCAGTYTTTTCAAC						
	*ankA*	ANK-F1	GAAGAAATTACAACTCCTGAAG	705	95/30	53/30	72/60	35	[10]
		ANK-R1	CAGCCAGATGCAGTAACGTG						
		ANK-F2	TTGACCGCTGAAGCACTAAC	664	95/30	52/30	72/60	5	
		ANK-R2	ACCATTTGCTTCTTGAGGAG		95/30	54/30	72/60	25	
*Borrelia* species	*pyrG*	pyrG-1F	ATTGCAAGTTCTGAGAATA	801	94/20	45/30	72/30	30	[11]
		pyrG-1R	CAAACATTACGAGCAAATTC						
		pyrG-2F	GATATGGAAAATATTTTATTTATTG	707	95/30	45/30	72/30	5	
					95/30	47/30	72/30	5	
		pyrG-2R	AAACCAAGACAAATTCCAAG		95/30	49/30	72/30	25	
*Bartonella* species	*ITS*	ITS_OF	TTCAGATGATGATCCCAAGC	639	95/30	48/30	72/60	5	[12]
		ITS_OR	AACATGTCTGAATATATCTTC		95/30	50/30	72/60	30	
		ITS_IF	CCGGAGGGCTTGTAGCTCAG	499	95/30	41/30	72/60	30	
		ITS_IR	CACAATTTCAATAGAAC						
*Coxiella burnetii*	*IS1111*	IS111F1	TACTGGGTGTTGATATTGC	485	95/15	52/5	72/30	35	[13]
		IS111R1	CCGTTTCATCCGCGGTG						
		IS111F2	GTAAAGTGATCTACACGA	260	95/15	56/15	72/15	30	
		IS111R2	TTAACAGCGCTTGAACGT						
*Babesia* species	18S rDNA	Bab5	AATTACCCAATCCTGACACAGG	485	94/60	55/60	72/120	35	[14]
		Bab8	TTTGGCAGTAGTTCGTCTTTAACA						
		Bab6	GACACAGGGGGTAGTGACAAGA	407	94/60	55/60	72/120	30	
		Bab7	CCCAACTGCTCCTATTAACCATTAC						
Ticks	16S rDNA	16S+1-F	CTGCTCAATGAATATTTAAATTGC	450	95/45	55/60	72/90	40	[15]
		16S+1-R	CGGTCTAAACTCAGATCATGTAGG						

^a^ *ompA*, outer membrane protein A gene; *gltA*, citrate synthase gene; *17 kDa*, 17 kDa protein gene; *groEL*, heat shock protein gene; *ankA*, ankyrin-related protein gene; *pyrG*, CTP synthase gene; *ITS*, 16S-23S internal transcribed spacer region; *IS1111*, htpAB-associated repetitive element; 18S rDNA, 18S ribosomal RNA gene; 16S rDNA, 16S ribosomal RNA gene.

The reaction mixture for N-PCR was identical to that used in C-PCR, except that the first PCR product was used as template DNA, and that N-PCR primers were included. For each PCR run, a positive and negative control (molecular grade water) were included. The detection limit of PCR used in this study was > 5 × 10^2^ copies/μL.

All amplifications were performed in an AB thermal cycler (Applied Biosystems, Foster City, CA, USA). The amplified products were separated via electrophoresis on a 1.2% agarose gel and stained with ethidium bromide for visualization.

### Sequencing and phylogenetic analysis

The amplified PCR products were purified using QIAquick PCR purification kits (QIAGEN, Hilden, Germany) and sequenced using PCR primers at Solgent Inc. (Daejeon, Korea). The sequences obtained in this study were compared to GenBank sequences using BLAST. Gene sequences, excluding the primer regions, were aligned using the multisequence alignment program in Lasergene version 8 (DNASTAR, USA). The nucleotide sequences obtained from the PCR amplifications performed in this study were registered and assigned the following GenBank accession numbers: Tick1 (MW481245) and Tick29 (MW481246) for the *ankA* gene; Tick29 (MW481247), Tick2 (MW481248), Tick17-1 (MW481249), Tick17-2 (MW481250), Tick15 (MW481251), Tick5 (MW481252), Tick6 (MW481253), Tick7 (MW481254), Tick18 (MW481255), Tick30 (MW481256), Tick19 (MW481257), Tick21 (MW481258), and Tick24 (MW481259) for the *gltA* gene; Tick25 (MW475155), Tick12 (MW475156), and Tick19 (MW475157) for the 18S rDNA.

Phylogenetic trees were constructed using ClustalW of the MegAlign Program (DNASTAR, USA) based on the alignments of positive gene sequences using the neighbor-joining method. Bootstrap analysis (1,000 replicates) was performed according to the Kimura 2-parameter method. Pairwise alignments were performed with an open-gap penalty of 10 and a gap extension penalty of 0.5.

## Results

### Tick identification

We obtained 33 ticks from 30 tick-bitten humans. Of these, 15 ticks (45.5%) were adults, namely 12 females and 3 males, and 18 ticks (54.5%) were nymphs. Based on morphological examination using a microscope, the ticks were identified as *Amblyomma testudinarium* (20, 60.6%; 7 adults and 13 nymphs), *Haemaphysalis longicornis* (9, 27.3%; five adults and four nymphs), and *Ixodes nipponensis* (4, 12.1%; three adults and one nymph), as described in Table 2. Tick identification using 16S rDNA C-PCR and DNA sequencing yielded the same results as the microscopic examination, except for four samples without tick DNA (Table 3). There were not enough tick lysates to extract DNA because we tried to isolate pathogens using the cell lines and mice.

**Table 2:** Developmental stages and species of ticks removed from tick-bitten humans determined from both morphological identification and 16S rDNA-targeting conventional PCR.

Tick species		*Amblyomma testudinarium*	*Heamaphysalis longicornis*	*Ixodes nipponensis*
Development stage	Adult female	4	5	3
	Adult male	3	0	0
	Nymph	13	4	1
	Larva	0	0	0
	Total No. (%)	20 (60.6 %)	9 (27.3 %)	4 (12.1 %)
		33 (100 %)		

**Table 3:** Characteristics of the 33 ticks using the DNA of tick-borne pathogens obtained from tick-bitten humans.

Patient no.	Patient age/sex	Tick species identified by a microscopy	Development stage (sex)	Tick engorgement	Identification of ticks by 16S rDNA PCR	Detected tick-borne pathogens in ticks		
						SFG *Rickettsia*	*A*. *phagocytophilum*	*Babesia* spp.
1	83/F	*A*. *testudinarium*	Nymph	+^a^	NA	-	*A*. *phagocytophilum*	-
2	46/M	*A*. *testudinarium*	Nymph	+	NA	*R*. *tamurae*	-	-
3	4/M	*A*. *testudinarium*	Nymph	*+*	*A*. *testudinarium*	-	-	-
4	NA	*A*. *testudinarium*	Nymph	NA^b^	*A*. *testudinarium*	-	-	-
5	65/M	*A*. *testudinarium*	Nymph	+	*A*. *testudinarium*	*R*. *tamurae*	-	-
6	74/M	*A*. *testudinarium*	Nymph	NA	*A*. *testudinarium*	*R*. *tamurae*	-	-
7	58/F	*A*. *testudinarium*	Nymph	NA	*A*. *testudinarium*	*R*. *tamurae*	-	-
8	52/F	*A*. *testudinarium*	Nymph	NA	*A*. *testudinarium*	-	-	-
9	62/F	*A*. *testudinarium*	Nymph	+	*A*. *testudinarium*	-	-	-
10	60/M	*A*. *testudinarium*	Nymph	+	*A*. *testudinarium*	-	-	-
11	55/F	*A*. *testudinarium*	Nymph	+	*A*. *testudinarium*	-	-	-
12	30/F	*A*. *testudinarium*	Nymph	+	*A*. *testudinarium*		-	*B*. *microti*
13	71/M	*A*. *testudinarium*	Nymph	+	*A*. *testudinarium*	-	-	-
14	64/M	*A*. *testudinarium*	Adult (female)	NA	NA	-	-	-
15	NA	*A*. *testudinarium*	Adult (female)	NA	*A*. *testudinarium*	*Ca*. R. jingxinensis	-	-
16	60/F	*A*. *testudinarium*	Adult (female)	+	*A*. *testudinarium*	-	-	-
17	54/F	*A*. *testudinarium*	Adult (female)	NA	*A*. *testudinarium*	*R*. *tamurae*	-	-
		*A*. *testudinarium*	Adult (male)	NA	*A*. *testudinarium*	*R*. *tamurae*	-	-
18	53/F	*A*. *testudinarium*	Adult (male)	+	*A*. *testudinarium*	*R*. *tamurae*	-	-
19	78/F	*A*. *testudinarium*	Adult (male)	+	*A*. *testudinarium*	*R*. *tamurae*	-	*B*. *gibsoni*
20	60/F	*H*. *longicornis*	Nymph	NA	*H*. *longicornis*	-	-	-
		*H*. *longicornis*	Nymph	NA	NA	-	-	-
21	83/F	*H*. *longicornis*	Nymph	NA	*H*. *longicornis*	*Ca*. R. jingxinensis	-	-
22	72/F	*H*. *longicornis*	Nymph	+	*H*. *longicornis*	-	-	-
23	76/F	*H*. *longicornis*	Adult (female)	+	*H*. *longicornis*	-	-	-
		*H*. *longicornis*	Adult (female)	+	*H*. *longicornis*	-	-	-
24	77/F	*H*. *longicornis*	Adult (female)	+	*H*. *longicornis*	*Ca*. R. jingxinensis	-	-
25	5/M	*H*. *longicornis*	Adult (female)	NA	*H*. *longicornis*	-	-	*B*. *microti*
26	54/F	*H*. *longicornis*	Adult (female)	NA	*H*. *longicornis*	-	-	-
27	77/F	*I*. *nipponensis*	Nymph	+	*I*. *nipponensis*	-	-	-
28	72/M	*I*. *nipponensis*	Adult (female)	NA	*I*. *nipponensis*	-	-	-
29	53/F	*I*. *nipponensis*	Adult (female)	+	*I*. *nipponensis*	-	*A*. *phagocytophilum*	-
30	81/F	*I*. *nipponensis*	Adult (female)	+	*I*. *nipponensis*	*R*. *monacensis*	*A*. *phagocytophilum*	-

^a^ Presence.

^b^ NA: not available.

#### Molecular detection of tick-borne pathogens in ticks removed from humans

We examined 33 ticks for the detection of tick-borne pathogens using pathogen-specific nested PCR. Tick-borne pathogens were detected in 16 ticks. From the results, 12 ticks (36.4%) were positive for spotted fever *Rickettsia* species, namely *R*. *monacensis* (1 of 33, 3.0%), *R*. *tamurae* (8 of 33, 24.2%), and *Candidatus* Rickettsia jingxinensis (3 of 33, 9.1%). Three ticks (9.1%) were positive for *A*. *phagocytophilum*, while the other three ticks (9.1%) were positive for either *B*. *gibsoni* (1 of 33, 3.0%) or *B*. *microti* (2 of 33, 6.0%) (Table 4). All ticks were negative for *Borrelia* spp., *Bartonella* spp., and *C*. *burnetii*.

**Table 4:** Detection of tick-borne pathogens in ticks via pathogen-specific nested PCR.

Detected pathogens	Positive tick numbers	/Total numbers	PCR positivity (%)
Spotted fever group *Rickettsia* species	**12**	/33	36.4
* R*. *monacensis*	1	/33	3.0
* R*. *tamurae*	8	/33	24.2
* Candidatus* Rickettsia jingxinensis	3	/33	9.1
*Anaplama phagocytophilum*	**3**	/33	9.1
*Babesia* species	**3**	/33	9.1
* B*. *gibsoni*	1	/33	3.0
* B*. *microti*	2	/33	6.0
*Borrelia* species	0	/33	0
*Bartonella* species	0	/33	0
*Coxiella burnetii*	0	/33	0

Of the three *A*. *phagocytophilum*-positive ticks, one tick was identified as *A*. *testudinarium*, and two ticks were identified as *I*. *nipponensis*. Of the 12 SFG *Rickettsia*-positive ticks, nine ticks were identified as *A*. *testudinarium*, two ticks were identified as *H*. *longicornis*, and one tick was identified as *I*. *nipponensis*. *R*. *tamurae* was identified only in *A*. *testudinarium*. Of the three ticks detected with *Babesia* spp., two were *A*. *testudinarium* and one was *H*. *longicornis*. Among the 33 ticks, one *I*. *nipponensis* (adult female) was co-infected with *A*. *phagocytophilum* and *R*. *monacensis*. In addition, co-infections of *R*. *tamurae* and *Babesia* spp. were identified in *A*. *testudinarium* (adult males), as presented in Table 3.

### Sequencing and phylogenetic analysis

The amplified PCR products were sequenced, and the sequencing results were aligned with the sequences obtained from the GenBank database to identify known sequences with a high degree of similarity using ClustalW. A neighbor-joining tree was constructed using the Kimura 2-parameter model (1,000 bootstrap replicates).

**Fig 1:**
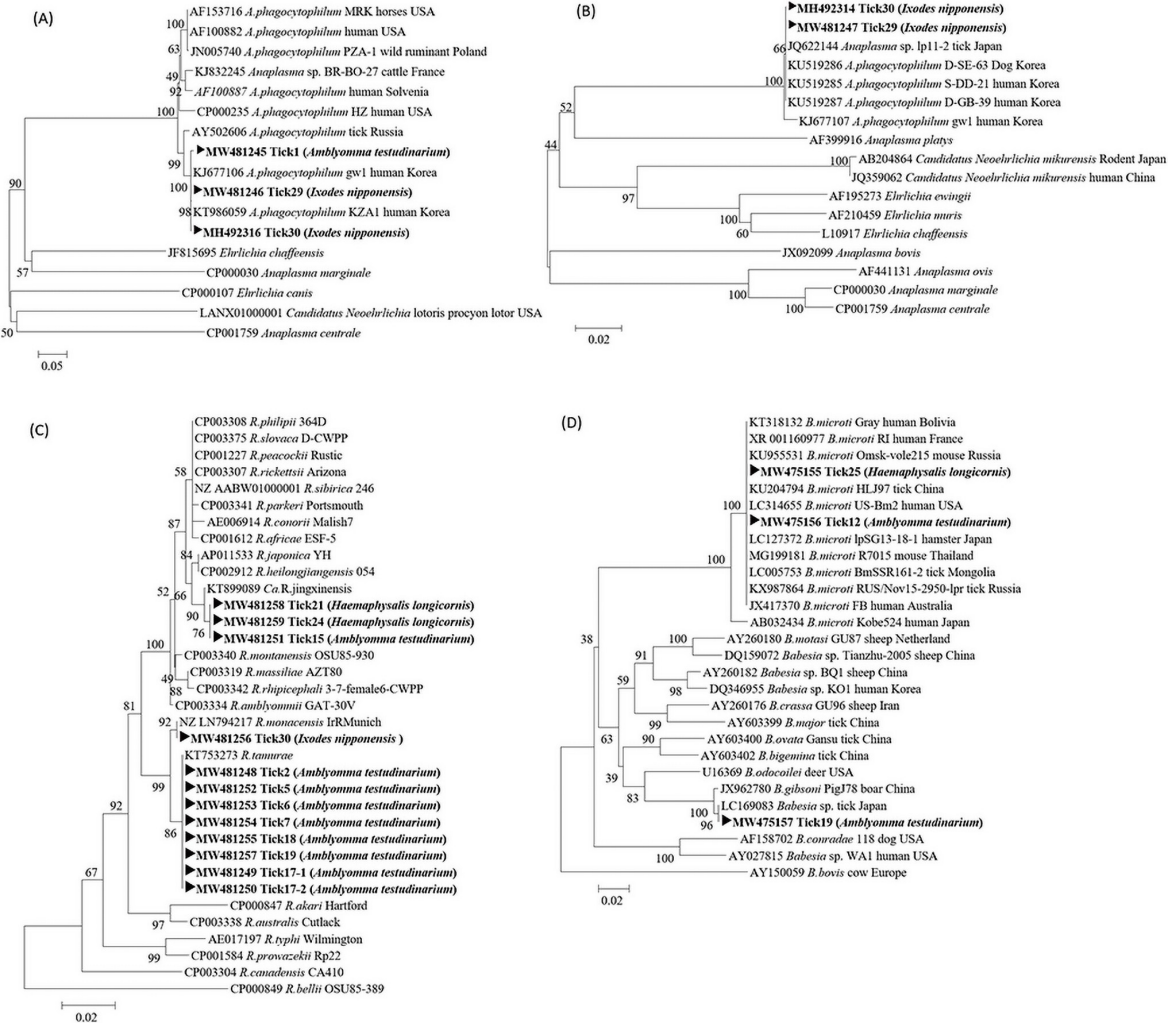
Phylogenetic trees based on partial nucleotide sequences obtained from *A*. *phagocytophilum-*, spotted fever group *Rickettsia-*, and *Babesia*-positive ticks in this study and from GenBank. (A) 560 bp portion of the *ankA* gene, (B) 330 bp portion of the *groEL* gene sequences for *A*. *phagocytophilum*, (C) 420 bp portion of the *gltA* gene sequences for SFG *Rickettsia*, and (D) 370 bp portion of the 18S rRNA gene sequences for *Babesia* species.

Partial *ankA* sequences obtained from *A*. *phagocytophilum*-positive ticks demonstrated 99% similarity with *A*. *phagocytophilum* (accession no. KJ677106 and KT986059, 98% bootstrap support; Fig 1A). The partial *ankA* sequences formed a cluster with *A*. *phagocytophilum* strains isolated from humans in the ROK. The partial *groEL* sequences obtained from *A*. *phagocytophilum*-positive ticks demonstrated 99% similarity with the *A*. *phagocytophilum* strain isolated from humans and dogs in the ROK (accession no. KU519286, 66% bootstrap support; Fig 1B).

The partial *17 kDa*, *ompA*, and *gltA* sequences obtained from SFG *Rickettsia-*positive ticks showed 99%–100% similarity with *R*. *tamurae*, *R*. *monacensis*, and *Ca*. R. jingxinensis. Phylogenetic analysis grouped partial *gltA* sequences with *R*. *tamurae* (accession no. KT753273, 86% bootstrap support; Fig 1C) and *R*. *monacensis* (accession no. NZ LN794217, 92% bootstrap support; Fig 1C), and *Ca*. R. jingxinensis (accession no. KT899089; 76% bootstrap support; Fig 1C).

The partial 18S rDNA sequences obtained from two *Babesia* species-positive ticks (Tick 12 and Tick 25) showed 99% similarity with a *B*. *microti* strain isolated from humans in the USA and a tick in China (accession no. KU204794 and LC314655, with 100% bootstrap support, Fig 1D). Another partial 18S rDNA sequence obtained from Tick19 had 99% similarity with *B*. *gibsoni* and 100% similarity with *Babesia* spp., which were clustered with a *B*. *gibsoni* strain isolated from a boar in China (accession no. JX962780, 100% bootstrap support, Fig 1D) and *Babesia* spp. from a tick in Japan (accession no. LC169083, 96% bootstrap support; Fig 1D).

## Discussion

Previous studies that investigated the prevalence of infectious agents in ticks collected by dragging and flagging grass vegetation in the ROK showed that *A*. *phagocytophilum* was detected in 1.9% of *H*. *longicornis* ticks [16] and 0.1% of *I*. *persulcatus* ticks, while *Rickettsia* spp. was detected in 1.7% of *H*. *longicornis* ticks [17]. One study reported that a pool of *H*. *longicornis*, *H*. *flava*, and *I*. *nipponensis* ticks collected by dragging vegetation in the ROK were positive for *Rickettsia* spp. 17 kDa antigen (60/311, 19.3%) and *ompA* (53/311, 17.04%) [18]. In the present study, the infection prevalence of *Rickettsia* species (*R*. *monacensis*, *R*. *tamurae*, and *Ca*. R. jingxinensis) and *A*. *phagocytophilum* in ticks collected from humans were higher than those of ticks collected from vegetation. Thus, we suggest that further studies be conducted to compare the infection prevalence of tick-borne pathogens, including *Rickettsia* spp., *A*. *phagocytophilum*, and *Babesia*, between ticks isolated from humans and ticks collected from grass vegetation.

*A*. *phagocytophilum* infection was first reported using serological evidence from humans in 2002, and it is currently the most frequently reported tick-borne bacterial infection in the ROK [19]. The detection of *Anaplasma* spp. in ticks from grazing cattle collected from all ROK provinces has been reported [20]. Another study confirmed the presence of human granulocytic anaplasmosis (HGA) caused by *A*. *phagocytophilum* in a patient from the ROK who had a history of tick bites, clinical symptoms, and positive laboratory findings [21]. The present results showed that *A*. *phagocytophilum* was detected in *A*. *testudinarium* and *I*. *nipponensis* ticks. The amplicon sequences of the partial *ankA* gene in *A*. *testudinarium* (Tick 1) and *I*. *nipponensis* (Tick 29 and Tick 30) showed > 99% similarity. In the phylogenetic analysis, the sequences of the *ankA* gene from different types of ticks clustered together showed > 99% similarity with *A*. *phagocytophilum* strains isolated from humans in the ROK (Fig 1A). In this study, of the 30 patients who were bitten by ticks, 8 patients showed systemic symptoms (26.7%) including fever, diarrhea, headache, general weakness and chill. In particular, we detected the same pathogen with *A*. *phagocytophilum* in Tick30 and the blood of these tick-bitten patients. It has been identified via PCR and increased antibody levels (IgG/IgM) using an indirect immunofluorescence assay according to the sample collection date of these patients [22]. Further study will need to be conducted to analyze patient characteristics from clinical data of more ticks and these tick-bitten patients.

The first isolation of *R*. *monacensis* from ticks in the ROK was reported in 2013 [23]. A previous study from the ROK reported that *I*. *nipponensis* was infected with the human pathogen *R*. *monacensis* and that *H*. *longicornis* and *H*. *flava* were infected with unknown SFG *Rickettsia* pathogens [18]. Our results confirmed the presence of *R*. *monacensis* in *I*. *nipponensis* ticks removed from humans. In addition, our results indicated that *I*. *nipponensis* ticks are most likely the vectors responsible for transmitting *R*. *monacensis* infections in the ROK. Therefore, further studies are needed to determine the role of *I*. *nipponensis* in the transmission of *R*. *monacensis* pathogens to humans, and that the blood of patients bitten by *I*. *nipponensis* ticks and the ticks themselves should be investigated for the presence of *R*. *monacensis*.

*R*. *tamurae* was first isolated from *A*. *testudinarium* ticks collected in Japan in 1993. *R*. *tamurae* was formally identified as a novel species via genetic and phylogenetic analyses in 2006 [24]. In 2011, its first case of human infection was confirmed via molecular and serological analyses in Japan [25]. The presence of SFG *Rickettsia*, including *R*. *tamurae*, was found in *Amblyomma* and *Dermacentor* ticks in Thailand [26] and in *Haemaphysalis* ticks in Peninsular Malaysia [27]. In addition, *R*. *tamurae* was found in *Amblyomma* ticks from an area endemic for Brazilian spotted fever in Brazil [28]. Supporting these previous studies, our results showed the presence of *R*. *tamurae* in *A*. *testudinarium* ticks.

The presence of potentially novel species of *Ca*. R. jingxinensis was proposed in *H*. *longicornis* nymphs from Jingxin in Northeastern China in 2016 [29] and was detected in *H*. *longicornis* ticks in Xi’an, China in 2017 [30]. However, in the ROK, the pathogenicity of *Ca*. R. jingxinensis is still unclear. Therefore, further assessment of its potential pathogenicity in humans and animals is warranted.

There have been no previous reports on *R*. *tamurae* or *Ca*. R. jingxinensis from ticks in the ROK. Here, we report the first identification of *R*. *tamurae* and *Ca*. R. jingxinensis from ticks obtained from tick-bitten humans. The discovery of new pathogens in ticks removed from humans suggests that the risk of disease outbreaks may increase in the future.

*Babesia* was first discovered in animals by Babes in 1988, and more than 100 species have been identified to date. In the ROK, *Babesia* spp. have been isolated from cattle and other mammals (raccoons, deer, and badgers) since the 2000s [31–33]. *Babesia* spp. are mainly carried by *Ixodes* ticks. Previous studies using ticks collected from grass and vegetation in the ROK reported that *H*. *longicornis* was the most common tick species infected with *Babesia* [17, 20]. Our results showed that *B*. *microti* was found in both *H*. *longicornis* and *A*. *testudinarium*. In the USA, the primary vector for the transmission of *B*. *microti* to humans is the tick *Ixodes scapularis* in the nymphal stage [34]. The present results suggest that further studies are needed to determine the type of ticks that are vectors for the transmission of *B*. *microti* to humans in the ROK.

*B*. *gibsoni* was first identified in the nymphs of *Rhipicephalus sanguineus* ticks from infected dogs in Asia [35]. In this study, *B*. *gibsoni* was detected in *A*. *testudinarium* ticks. The first case of human babesiosis (KO1) was reported in 2007 in the ROK and was highly related to Chinese ovine *Babesia* spp. [14]. Based on the phylogenetic analysis of the 18S rDNA gene in our study, the pathogen clustered with a group of *Babesia* spp. isolated from a tick in Japan, which diverged from the KO1 strain (Fig 1D). The present results indicate that *Babesia* spp. may vary according to their geographical distribution.

This study was limited because only a few samples were obtained from the southwestern area of the ROK. Chosun University Hospital is located in Gwangju Metropolitan City, a province in the southwest of the ROK, and patients who visit this hospital are mainly residents of Gwangju Metropolitan City and Jeollanam-do province, which are located nearby (S1 Fig). Therefore, the sampling site of ticks removed from tick-bitten humans is limited to the southwestern region of the ROK. Further studies should be conducted to investigate pathogen prevalence in ticks removed from tick-bitten humans in more areas. Such studies could enable the identification of geographic regions with a high risk of tick-borne diseases. Additionally, further investigation is needed to determine the difference between pathogens found in ticks isolated from humans and ticks collected from grass vegetation. In addition, transmission studies should be conducted to determine whether the pathogens found in ticks are the same as those found in humans bitten by ticks. Serological testing of the blood of tick-bitten patients and ticks will be necessary to confirm the transmission of pathogens from ticks to humans. A study has reported the seroprevalence of *A*. *phagocytophilum* in human populations analyzed based on global data published from 1994 to 2018. Its seroprevalence was highest in the high-risk population (13.8%) and lowest in the healthy population (5.0%). The estimated seroprevalences of *A*. *phagocytophilum* in febrile patients, tick-bitten, and tick-borne disease populations were 6.4%, 8.0%, and 9.0%, respectively [36]. A serological study of *R*. *japonica* showed 19.88% seropositivity in 3,401 patients with acute febrile illness, and another reported 8% *R*. *sibirica* seropositivity and 14.34% *R*. *coronii* seropositivity in 3,362 patients with the same condition [37, 38]. Further experiments and correlation analyses using blood samples from tick-bitten humans and ticks isolated from them may help predict the transmission of tick-borne diseases.

In conclusion, we confirmed the presence of three tick species carrying tick-borne pathogens, the most common of which was *A*. *testudinarium*, followed by *H*. *longicornis* and *I*. *nipponensis*. These ticks were positive for SFG, *Rickettsia*, *A*. *phagocytophilum*, and *Babesia*. To our knowledge, this is the first report of the presence of *R*. *tamurae* and *Ca*. R. jingxinensis in ticks removed from tick-bitten humans in the southwestern area of the ROK.

## Supporting information

S1 Fig Location surveyed for ticks from tick-bitten patients in the southwest provinces of the ROK. Dark red color, Gwangju Metropolitan City and Chosun University Hospital located in this area; light red color, Jeollanam Provinces.
(PDF)
