# Supplementary material for: Molecular investigation of tick-borne pathogens in ticks removed from tick-bitten humans in the southwestern region of the Republic of Korea
Source: PLoS One. 2021 Jun 15;16(6):e0252992. doi: 10.1371/journal.pone.0252992 (PMC8205137; doi:10.1371/journal.pone.0252992)
Supplement: S1 Fig — Dark red color, Gwangju Metropolitan City and Chosun University Hospital located in this area; light red color, Jeollanam Provinces. (PDF) [file pone.0252992.s001.pdf]

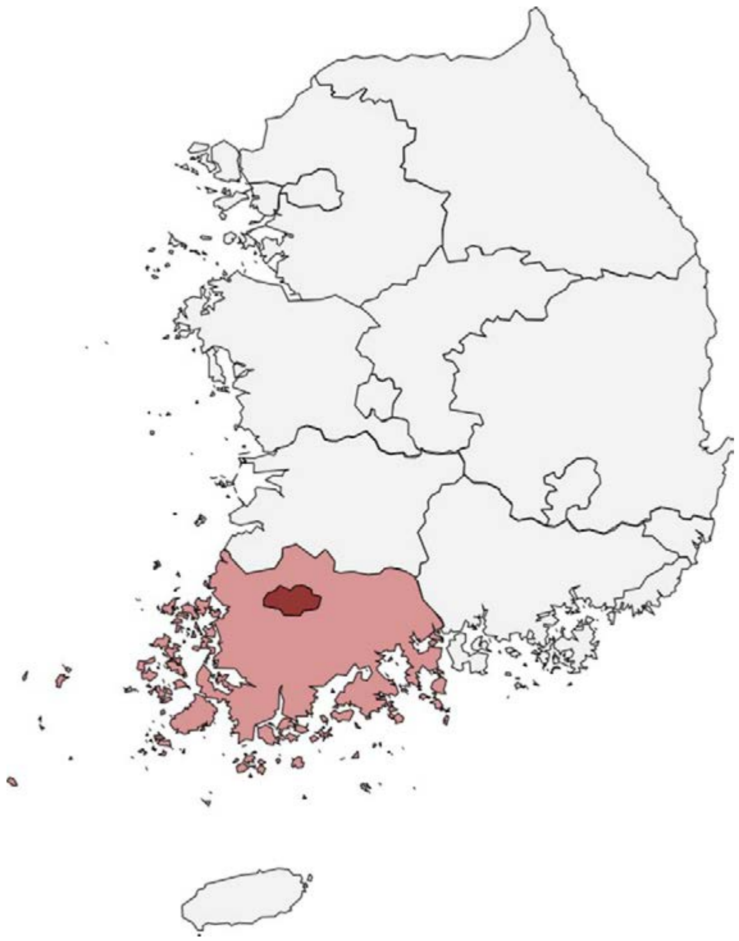

S1 Fig. Location surveyed for ticks from tick-bitten patients in the southwest provinces of the ROK. Dark red color, Gwangju Metropolitan City and Chosun University Hospital located in this area; light red color, Jeollanam Provinces
